# MRS and DTI evidence of progressive posterior cingulate cortex and corpus callosum injury in the hyper-acute phase after Traumatic Brain Injury

**DOI:** 10.1080/02699052.2019.1584332

**Published:** 2019-03-08

**Authors:** Tim P. Lawrence, Adam Steel, Martyn Ezra, Mhairi Speirs, Pieter M. Pretorius, Gwenaelle Douaud, Stamatios Sotiropoulos, Tom Cadoux-Hudson, Uzay E. Emir, Natalie L. Voets

**Affiliations:** a FMRIB Centre, Wellcome Centre for Integrative Neuroimaging, Nuffield Department of Clinical Neurosciences, University of Oxford, Oxford, United Kingdom; b Department of Neuroscience, Oxford University Hospitals NHS Foundation Trust, Oxford, United Kingdom; c Laboratory of Brain and Cognition, National Institute of Mental Health, National Institutes of Health, Bethesda, MD, USA; d Sir Peter Mansfield Imaging Centre, School of Medicine, University of Nottingham, Nottingham, UK; e National Institute for Health Research (NIHR) Nottingham Biomedical Research Centre, Queens Medical Centre, Nottingham, UK; f School of Health Sciences, Purdue University, West Lafayette, IN, USA

**Keywords:** Magnetic resonance imaging, diffuse axonal injury, secondary injury cascade, corpus callosum, posterior cingulate cortex

## Abstract

The posterior cingulate cortex (PCC) and corpus callosum (CC) are susceptible to trauma, but injury often evades detection. PCC Metabolic disruption may predict CC white matter tract injury and the secondary cascade responsible for progression. While the time frame for the secondary cascade remains unclear in humans, the first 24 h (hyper-acute phase) are crucial for life-saving interventions.

**Objectives**: To test whether Magnetic Resonance Imaging (MRI) markers are detectable in the hyper-acute phase and progress after traumatic brain injury (TBI) and whether alterations in these parameters reflect injury severity.

**Methods**: Spectroscopic and diffusion-weighted MRI data were collected in 18 patients with TBI (within 24 h and repeated 7–15 days following injury) and 18 healthy controls (scanned once).

**Results**: Within 24 h of TBI N-acetylaspartate was reduced (F = 11.43, p = 0.002) and choline increased (F = 10.67, p = 0.003), the latter driven by moderate-severe injury (F = 5.54, p = 0.03). Alterations in fractional anisotropy (FA) and axial diffusivity (AD) progressed between the two time-points in the splenium of the CC (p = 0.029 and p = 0.013). Gradual reductions in FA correlated with progressive increases in choline (p = 0.029).

**Conclusions**: Metabolic disruption and structural injury can be detected within hours of trauma. Metabolic and diffusion parameters allow identification of severity and provide evidence of injury progression.

## Introduction

Traumatic brain injury (TBI) is a leading cause of mortality and morbidity worldwide. Gaps in our knowledge regarding the pathophysiology of the secondary injury cascade in the first few hours following trauma limit our ability to accurately classify patients and optimize treatment. Biochemical and metabolic markers that reflect the broad pathophysiological heterogeneity and injury progression following TBI could potentially guide clinical decisions regarding hospital admission, escalation of care and therapeutic interventions in the hyper-acute phase (<24 h) following trauma.

Studies investigating all severities of injury following TBI highlight a crucial link between diffuse axonal injury (DAI) and patients’ long-term clinical outcome and persistent deficits (1–3). DAI results from rapid acceleration/deceleration of the head, causing stretch and shear injuries to axons and blood vessels. The viscoelastic nature of white matter axons makes them particularly susceptible to damage (4,5). Axonal injury results not just from the initial mechanical insult (primary injury) but due to a progressive molecular and cellular cascade of pathologic changes within the axon after the initial stress (4,5) causing secondary injury detectable using DTI in a lateral fluid percussion (LFP) model in rats (6).

DAI is now considered a process influencing multiple disparate brain regions (7). The secondary injury cascade is thought to be responsible for the progression of DAI with membrane disruption, excitotoxicity, inflammation and neuronal injury affecting the evolution of axonal pathology (3,8,9). Despite attempts using LFP models in rats to better understand the secondary cascade in white matter tracts questions remain regarding the microstructural disturbance that occurs after trauma (6). MRI techniques, and in particular Magnetic Resonance Spectroscopy (MRS), have identified clinically relevant injury patterns in the first few days after trauma (10,11). Specific metabolites provide information regarding separable pathophysiological processes occurring in the primary and secondary injury phases following TBI (12). Decreased *N*-acetylaspartate (NAA), a marker of neuronal density and viability, and increased choline (Cho), a marker of membrane synthesis and gliosis have been demonstrated in the acute phase (days) (13). Additionally, early excitotoxic injury (within 7 days) has been indicated by elevated glutamate/glutamine (Glx) in white matter (14), which conversely, may be reduced in the gray matter following TBI (15,16).

MRS evidence of diffuse injury in TBI has been hypothesized to reflect differential processes across injury severities (17–19). Kirov hypothesized that mild, as well as severe TBI, can result in DAI with cell dysfunction resulting from mild injury and cell death and Wallerian degeneration resulting from more severe injury (18). Empirical MRI evidence differentiating early secondary injury processes among TBI severities, however, has been limited by practical and clinical challenges hindering the investigation of patients with severe injury in the first few hours after trauma. In animal studies, early microstructural and metabolic changes following controlled cortical impact injury indicate that there may be an optimal temporal window (within the first 4 h) for interventions that could limit secondary damage (12). In humans, metabolite abnormalities provide correlates of injury severity and long-term clinical outcome (1,2,13). However, the critical time window of biochemical disruption underpinning the development of DAI has not yet been defined. Animal studies using LFP techniques have highlighted the application of diffusion-weighted imaging in investigating microstructural injury and its correlation with cellular level alterations (6). Recently, through the emergence of dedicated acute brain injury MRI centres, equipped to image severely injured intubated and ventilated patients, it has become possible to study pathophysiological changes occurring in the immediate aftermath of severe human TBI.

The grey-white matter junction and midline brain structures have been reported as particularly vulnerable in TBI. The corpus callosum (CC), a white matter tract critical for interhemispheric communication, is reported to be frequently impacted (20,21) and CC damage could offer an anatomical imaging correlate of injury severity (22). Injury to the posterior cingulate cortex (PCC), a highly anatomically connected region forming part of the posterior medial cortex (23), has also been shown to predict outcome following TBI (24,25). The PCC plays an important role in health and disease, and abnormal function following TBI is thought to result in attentional deficits (26). The PCC is bounded superiorly by the marginal ramus of the cingulate sulcus, posteriorly by the parieto-occipital sulcus, anteriorly by Brodmann area 24 and inferiorly by the corpus callosum (26). The cingulum bundle (CB) provides structural connection between the PCC, the medial temporal lobes and the ventromedial prefrontal cortex (27). Damage to functional networks that converge on midline areas vulnerable to TBI are thought to be associated with persistent post-traumatic complaints (28). Alterations in imaging parameters within inter-related cortical and white matter regions susceptible to injury may, therefore, shed light on the metabolic disruption that underpins DAI. To the best of our knowledge, the relationship between metabolic disruption in cortical regions and injury of white matter tracts that link them at a network level has not been investigated, in humans, in the first 24 h following TBI.

Diffusion-weighted imaging provides the primary method to non-invasively detect and characterize axonal injury in the living human brain (29–31). Diffusion tensor imaging (DTI) maps the directionality and rate of diffusion of water molecules within tissue. The basis and considerations surrounding DTI have been extensively reviewed (32). For every imaged voxel in the brain, DTI can provide a range of tissue microstructure estimates. Fractional anisotropy (FA) quantifies the fraction of diffusion that is anisotropic and provides information about local fibre coherence. Mean diffusivity (MD) is used to quantitatively assess the magnitude of diffusion independent of direction and is considered to be an inverse measure of membrane density. Axial diffusivity (AD) represents diffusivity along the principal axis and, in white matter tracts, is considered to be parallel to axonal fibres. Radial diffusivity (RD) is the apparent water diffusion coefficient in the directions perpendicular to the fibres (33). These measures reflect the cellular environment and have been shown to be sensitive indicators of axonal injury in TBI (34).

FA has been shown to decrease (35) and MD increase in patients with TBI imaged in the subacute to chronic phase following injury (36–38). But there is a paucity of information surrounding axonal injury in the hyper-acute phase (<24 h) in human TBI. Animal studies of severe TBI using DTI and histological analysis have revealed a temporal pattern of variation in diffusion parameters providing insight into the underlying pathologies reflecting varying injury states over time (39–42). Reduced FA, AD and MD provided evidence of axonal damage within 24 h of injury, confirmed histologically with further evidence of reactive gliosis by 4 days. After a week macrophage infiltration, demyelination and edema was associated with elevated RD, AD and MD and decreased FA (41). Although the timing of injury progression has been demonstrated in animal models (43), in humans the equivalent temporal cascade remains unclear.

Here, we set out to characterize the time frame of cortical and white matter injury progression across the hyper-acute and acute phases in patients with TBI. We acquired MRSI and DTI in patients with TBI and age-matched healthy controls to test the following two hypotheses. Firstly, that metabolic disruption in the posterior cingulate cortex, and white matter injury to the corpus callosum and cingulum bundle, are detectable within the first few hours of trauma and progress during the first week. Secondly, on the assumption that the metabolic disruption represents the diffuse secondary cascade which contributes to the evolution of DAI, we hypothesized that metabolic disruption and microstructural injury processes are correlated with each other and reflect initial severity and clinical outcome at 1 week.

## Methods

### Participants

Eighteen adults with a range in severity of TBI (mean age: 48 years, range: 23–83, 13 men, 5 women) were recruited prospectively from the Emergency Department of the Oxford University Hospitals NHS Foundation Trust (Table 1). All patients received a CT scan as part of the standard trauma imaging protocol (according to the NICE head injury guidelines (44)). Once life-threatening injuries were ruled out, patients were recruited for the study. Exclusion criteria included contraindications to MRI, other injuries leading to instability that would compromise patient safety in the scanner, and requirement for urgent surgery. MRI scans were performed within 24 h and repeated again at 7–15 days following injury. In one patient, surgical intervention (fixation) for an upper limb fracture precluded repeat imaging at 7 days on safety grounds. The data from this participant were, therefore, only available from the first time-point. Clinical assessments, including GCS and neurological examination, were made throughout the study time period. Initial severity of injury was assessed using post-resuscitation GCS at presentation. Severity at one week/short term outcome was assessed using GCS at 7 days post injury. Patients with a GCS of 12 or less were considered moderate-severe. Patients with a GCS≥13 were considered mild. All patients with a GCS 12 or less and/or still in hospital (due to the TBI) at 7 days were considered moderate-severe at this point. Eighteen healthy controls age matched to the TBI group (mean age 41, range: 24–76, independent samples t-test t = −1.34, p = 0.19, 11 women, with no neurological or psychiatric history, nor any history of significant head injury) were also recruited. None of the participants received anti-coagulants, blood transfusions or clotting factors during the study period.

**Table 1.** Patient demographics.

Patient	Age	Gender	Initial GCS	GCS at scan 2	Intracranial injuries	Extracranial injuries	Time to scan 1 (hours)	Time to scan 2 (days)	Discharged from hospital by 1 week
1	36	Male	7	15	IVH (seizure)	Pneumothorax, scalp haematoma	21	8 days	Yes
2	83	Male	6	Intubated and sedated	ASDH	-	3	7 days	No
3	22	Male	14	15	-	-	12	7 days	Yes
4	42	Male	14	15	-	-	18	7 days	Yes
5	70	Female	15	15	-	-	3	15 days	Yes
6	44	Male	8	15	(seizure)	-	15	9 days	No
7	51	Male	15	15	-	-	3.5	7 days	Yes
8	30	Male	14	15	-	-	5	X	Yes
9	61	Female	15	15	EDH	-	21	7 days	Yes
10	42	Female	14	15	-	-	3.5	7 days	Yes
11	25	Female	15	15	-	-	4	7 days	Yes
12	30	Male	14	15	-	-	15	7 days	Yes
13	52	Female	3	15	IVH, TSAH, ASDH	-	6	7 days	Yes
14	41	Male	14–8	Intubated and sedated	Frontal and temporal contusions	Rib fractures, cervical and thoracic spinal fractures, wrist fracture	6	11 days	No
15	62	Male	12	15	-	Facial fractures	3.5	7 days	Yes
16	55	Male	12	15	-	Facial abrasions, lacerations and fractures	6.5	14 days	Yes
17	72	Male	3	Intubated and sedated	Multiple bilateral contusions, TSAH, pneumocephalus, petechial haemorrhages	Shoulder abrasions, skull base fractures, facial fractures	5.5	12 days	No
18	48	Male	10	Intubated and sedated	TSAH, contusions, petechial haemorrhages,	Cervical fracture, eye injury, wrist clavicle and rib fractures, extensive skull fractures frontal sinus and orbit fractures, skull base fracture	5	12 days	No

GCS = Glasgow Coma Scale, IVH = intraventricular haemorrhage, ASDH = acute subdural haematoma, EDH = extradural haematoma, TSAH = traumatic subarachnoid haemorrhage.

All patients with capacity at the time of initial recruitment gave written informed consent. For patients lacking capacity, the lead clinician, in consultation with the family, signed a declaration form to confirm agreement for the patient to be recruited into the study. Capacity was assessed by the lead clinician caring for the patient. Patients with a GCS of 15 who were orientated in time and place were deemed to have capacity if it was felt they had the ability to use and understand information to make a decision regarding involvement in the study, and communicate any decision made.

Explicit patient consent was sought as soon as possible upon recovery. Healthy controls provided informed written consent. The study was approved by the South Central – Berkshire Research Ethics Committee.

### MRI data acquisition

**Figure 1.**
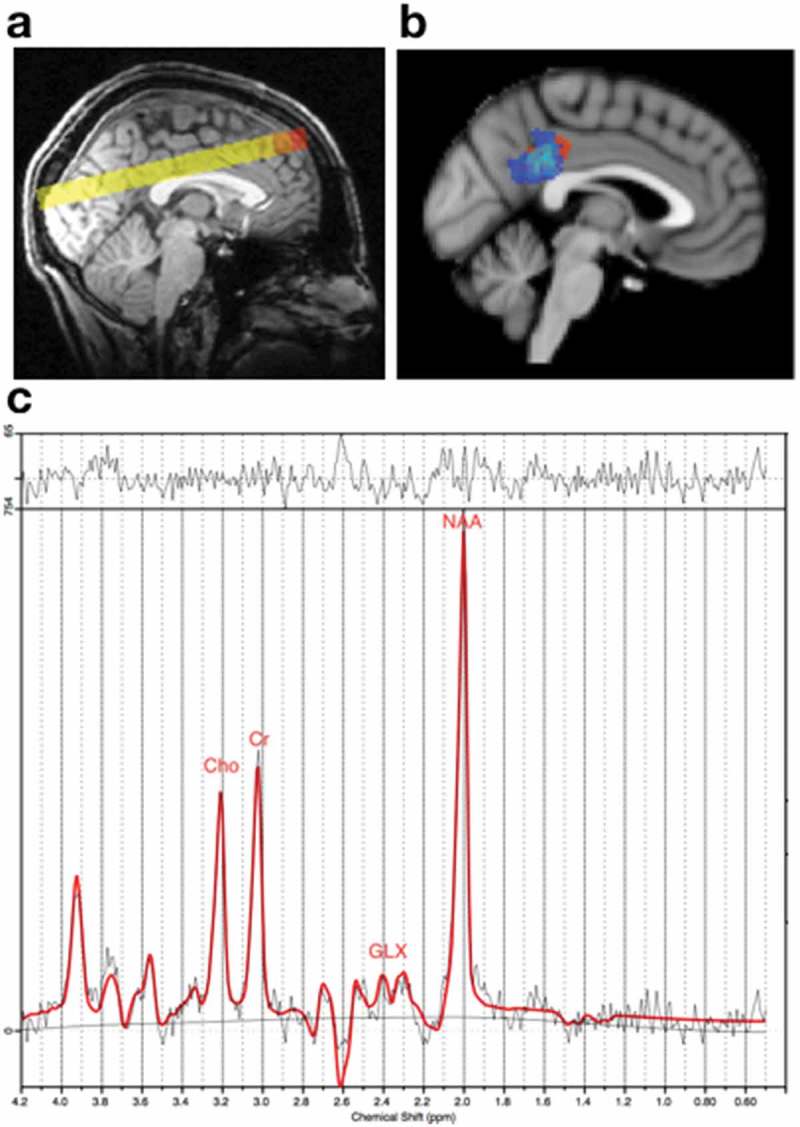
2D CSI slab and example spectra. (a) T1 from a representative subject showing the position of the 2D CSI slab. b) Overlap map showing posterior cingulate cortex voxels selected for analysis from the 2D CSI slab for each subject overlaid onto the Montreal Neurological Institute (MNI) template brain. (c) A representative spectrum from a healthy control displaying peaks for total choline (tCho), total creatine (tCr), glutamate and glutamine combined (GLX) and total N-acetyl aspartate (tNAA). No traumatic lesions, visible on structural imaging, were seen within the posterior cingulate cortex.

All imaging data were acquired on a 3T Siemens Magnetom Verio scanner at the Oxford Acute Vascular Imaging Centre (AVIC). The scanning protocol included T1-weighted MPRAGE, T2-weighted Turbo spin echo and T2* susceptibility weighted structural imaging (SWI) sequences, 2D proton magnetic resonance spectroscopic (MRS) chemical shift imaging (CSI) and diffusion-weighted imaging. SWI findings for a subset of this study population have been reported previously (45). Single-slab multi-voxel MRSI data were acquired using a point-resolved spectroscopy (PRESS) sequence using the following parameters: TR = 1700 ms, TE = 135 ms. Six saturation bands were applied (40 mm superior and inferior, 50 mm anterior, posterior, right and left lateral). A single image slab of 160mm^3^ x 160mm^3^ x 15mm^3^ was placed, according to atlas identified anatomical landmarks, to include the posterior cingulate cortex superior and posterior to the splenium of the corpus callosum (Figure 1). This MRSI slab consisted of a 160 mm x 160 mm grid containing 256 voxels each with a resolution of 10mm^3^ x 10mm^3^ x 15mm^3^. The cortical site of injury never extended into the MRS voxels used in the analysis (the location of the posterior cingulate cortex). Diffusion-weighted images were acquired along 64 diffusion directions at a b-value of 1500/mm^2^ with 5 additional b = 0 measures, using a voxel size of 2 × 2 x 2 mm, TR = 2153 ms, TE = 85 ms. A simultaneous multi-slice acquisition protocol (multiband factor 2) (46) was used to acquire two full diffusion data acquisitions in opposing phase-encode directions (Anterior–Posterior and Posterior–Anterior, for the purpose of image distortion correction, described below).

### Image processing – magnetic resonance spectroscopy

The MRSI slab was reconstructed in each subject’s native space by linearly registering the acquired MRSI slab to the participant’s T1 scan. Although efforts were made to place the MRSI slab in a matching location for all subjects, the exact slab location naturally varied due to each participant’s head position in the scanner and anatomical variation between participants (Figure 1). No traumatic lesions, visible on structural imaging, were seen within the posterior cingulate cortex. Two factors with high bearing on the reliability of metabolite interpretations are; consistency in the anatomical area measured across participants, and the reliability of metabolite concentrations as determined by Cramer Rau Lower bounds (CRLB). In order to maximize consistency in the cingulate cortex area sampled between subjects, and for longitudinal comparison within subjects, we identified a common ‘core’ subset of voxels for analysis. The selected voxels provided the highest degree of anatomical consistency between participants and the highest quality spectral data. First, we excluded all peripheral voxels in the MRSI slabs, which were located within the saturation bands. From the remaining central core of each participant’s MRSI slab, two voxels were selected based firstly on anatomical location (immediately posterior and superior to the splenium of the corpus callosum) and secondly, on spectral quality (CRLB values >25% were discarded).

Metabolite concentration ratios were used relative to total creatine (tCr) (creatine + phosphocreatine). Ratios were generated from total (t) metabolite values of N-acetylaspartate + N-acetylaspartylglutmate (NAA + NAAG = tNAA), phosphocholine + glycerophosphocholine (PCho + GPC = tCho) and Glutamate + Glutamine (GLX). tNAA/tCr, tCho/tCr, and Glx/tCr, were estimated using LC Model software (http://www.s-provencher.com/pages/lcmodel.shtml). Voxels with metabolite concentration standard deviations (CRLB) >25% were considered not reliable and were not selected for analysis. Based on this criteria, one patient with TBI and two healthy controls were excluded from metabolite analyses. Metabolite concentrations were expressed as ratios relative to tCr to control for between-voxel variation in absolute concentrations. The ratio values were multiplied by 8 within the LC Model to account for the estimated concentration of normal tCr, as previously described (47). tCr absolute concentration did not differ between controls and patients (independent samples t-test t = −1.12, p = 0.28).

In order to adjust metabolite ratio measurements for the amount of grey matter within the selected voxels, voxel-wise grey matter probability values were calculated from each participant’s T1-weighted anatomical scan using FMRIB’s Automated Segmentation Tool (FAST) (48). Following segmentation into grey matter, white matter and cerebrospinal fluid (CSF) tissue types, FAST calculates a partial volume estimate (PVE) for each voxel providing the best estimate of the proportion of grey matter in that voxel. These PVE values, averaged across both voxels, were included in our metabolite-based statistical analyses as covariates.

### Image processing – diffusion tensor imaging

DTI data were pre-processed using FSL tools (49) (https://fsl.fmrib.ox.ac.uk/fsl/fslwiki/FDT/UserGuide). Image distortions associated with magnetic field inhomogeneities in echo-planar imaging sequences were corrected using the tool ‘topup’ (50). Data were collected with reversed phase encode blips to estimate the susceptibility induced field based on the five non-diffusion weighted images acquired during the anterior-to-posterior and the posterior-to-anterior phase encode acquisitions. From these pairs of images, with distortions going in opposite directions, the susceptibility induced off-resonance field was estimated (49,51). The resulting fieldmap was fed into the tool ‘eddy’ to simultaneously correct the diffusion-weighted images for susceptibility-induced distortions, eddy current-induced distortions and head movement (50). Finally, diffusion tensors were fit to the data in order to derive fractional anisotropy (FA), mean diffusivity (MD), axial diffusivity (AD), lambda 2 and lambda 3 (λ2 and λ3) maps. The mean of λ2 and λ3 was calculated to generate radial diffusivity (RD).

Voxel-wise analyses of diffusion parameters (FA, AD, RD and MD) were performed using Tract-Based Spatial Statistics (TBSS) (52). This well-validated technique permits voxel-wise comparison of diffusion metrics by generating, through nonlinear registration to a standard FA template, a white matter “skeleton” that represents the core white matter within a study population, and then sampling individual-subject diffusion parameters onto this skeleton for statistical analysis (52). In this way, TBSS attempts to overcome difficulties both in aligning white matter across subjects and with spatial smoothing, resulting in improved sensitivity, objectivity and interpretability of analysis of multi-participant studies (49,52,53). The TBSS-derived skeleton was thresholded at 0.3 before FA, AD, RD and MD values for individual participants were projected onto the skeleton. In order to restrict our statistical analyses to voxels within the cingulum bundle (CB) and corpus callosum (CC), these two regions of interest were extracted from the probabilistic Harvard-Oxford parcellation atlas, formed from manual segmentation of the T1-weighted images acquired from 37 healthy volunteers performed by the MGH Centre for Morphometric Analysis. The atlas-derived masks were thresholded in order to retain only those voxels common to at least 95% of the atlas population to minimize co-inclusion of neighbouring structures.

### Longitudinal TBSS analysis

Three TBSS analyses were performed to assess; a) microstructural tissue properties in the corpus callosum in patients compared with controls at time point 1 (within 24 h) and again at time point 2 (7–15 days after injury), b) evidence of evolution of injury between time point 1 and 2 within patients, and c) a relationship between metabolite ratios in the posterior cingulate cortex and the diffusion parameters along the corpus callosum + cingulum bundles. To test for *evolution* in diffusion parameters between scan 1 and scan 2, a longitudinal TBSS method was used to minimize potential registration biases between data acquired at different time points. In this longitudinal approach (previously detailed (54)), the *difference* in FA between time point 1 and time point 2 was analysed, using a population-optimized skeleton. In brief, we used the FSL tool SIENA, applied to the non-diffusion weighted reference images for each patient to generate a halfway space between the images acquired during scan 1 and scan 2 (55). The FA maps for scan 1 and scan 2 were subsequently registered into halfway space for each participant using the transformation matrices generated by SIENA and then averaged (by summing the maps together and dividing by 2). An optimized study-specific skeleton was generated, based on the newly-created averaged FA maps, in order to reduce possible registration (and parameter sampling) biases that might arise if the skeleton was created based only on the images of scan 1 or of scan 2. Next, we created individual-subject FA difference maps by subtracting scan 2’s halfway-space FA map from scan 1’s halfway-space FA map. Finally, the FA difference values were projected onto the study-specific skeleton and permutation tests (5000 permutations) were performed to test for a linear trend over time (i.e. to test for a positive (or a negative) deviation from zero in the FA change between the two visits). The same analyses were repeated for AD, RD and MD.

### Statistical analysis

Statistical analyses were performed using SPSS version 24. Independent samples t-tests were performed to determine any differences in age and grey matter probability between controls and patients. Multivariate analyses of covariance (MANCOVA) were performed to compare the metabolite ratios i) between the controls and patients at scan 1 and scan 2 and ii) between patient severity sub-groups (measured by initial GCS and GCS plus hospital admission status at 1 week). Paired sample t-tests were performed to compare metabolite parameters in patients between scan 1 and scan 2. Both age and GM were included as covariates of no interest in the analyses of metabolite concentrations. Voxel-wise analyses of diffusion-based white matter microstructure parameters were performed through a 2D implementation of permutation testing within FSL “randomise”, including age as a nuisance regressor. Randomize permits permutation-based nonparametric inference testing within the framework of the general linear model. Group-wise comparisons (controls versus patients with TBI) were performed using two-sample unpaired t-tests, while relationships between metabolite ratios and diffusion parameters were identified by including de-meaned metabolite concentrations as covariates in the general linear model submitted to permutation testing. Linear effects of time were assessed using a single group paired t-test. Threshold-Free Cluster Enhancement (TFCE) was implemented to identify clusters with p-values less than 0.05 (family-wise error corrected for multiple comparisons). Due to the exploratory nature of the study, correlation analyses were not corrected for multiple comparisons.

## Results

### Age and grey matter

No significant difference was found in age (t = −1.34, p = 0.19) and mean posterior cingulate cortex grey matter probability estimates (t = −0.17, p = 0.87) between healthy controls and patients with TBI.

### Metabolite disruption and evolution

No traumatic lesions, identifiable on structural imaging (CT and MRI T1-weighted) were visible within the anatomical region of the voxels analysed in the posterior cingulate cortex.

**Figure 2.**
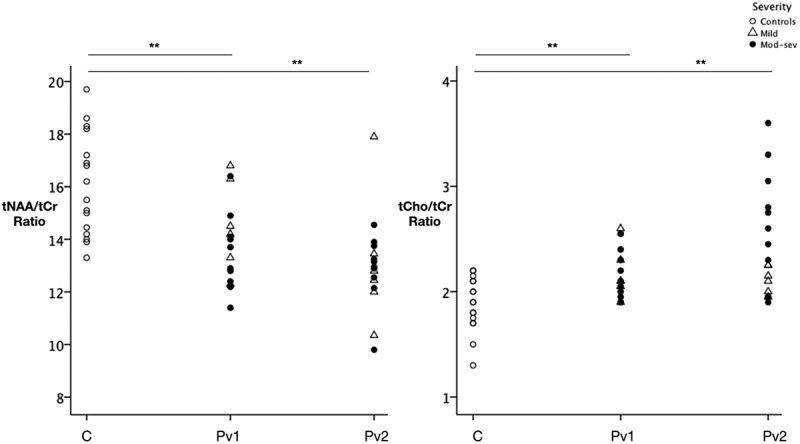
Metabolite ratios in controls and TBI patients within 24 h of injury and after 7 days. Scatter plots displaying the metabolite ratios of a) total N-acetyl aspartate (tNAA) to total creatine (tCr) ratio and b) total choline (tCho) to total Cr ratio. a) The tNAA/tCr ratio was lower in patients compared with controls at both visits (scan 1: F = 11.43 p = 0.002, scan 2: F = 8.81 p < 0.001. b) tCho/tCr ratios were significantly higher in TBI patients when compared to controls at both scan 1 (F = 10.67 p = 0.003) and scan 2 (F = 10.47 p = 0.001). C = Controls, Pv1 = Patient visit 1, Pv2 = Patient visit 2. ** represents a value for p < 0.001.

In the hyper-acute phase (within 24 h of injury) the tNAA/tCr ratio in the posterior cingulate cortex (PCC) was 15% lower in patients than in healthy controls (F = 11.43, p = 0.002) while the tCho/tCr ratio was 10% higher in patients compared with controls (F = 10.67, p = 0.003) (Figure 2a and b). No significant difference in GLX/tCr ratio was seen at this time. In the acute phase (7–15 days), tNAA/tCr ratio in the PCC remained lower in patients than in controls (on average 20% lower, F = 8.81, p < 0.001). TCho/tCr ratio remained elevated in patients compared with controls (on average 23%, F = 10.47, p = 0.001) (Figure 2a and b). No significant difference was seen in GLX/tCr ratio at 7 days.

**Figure 3.**
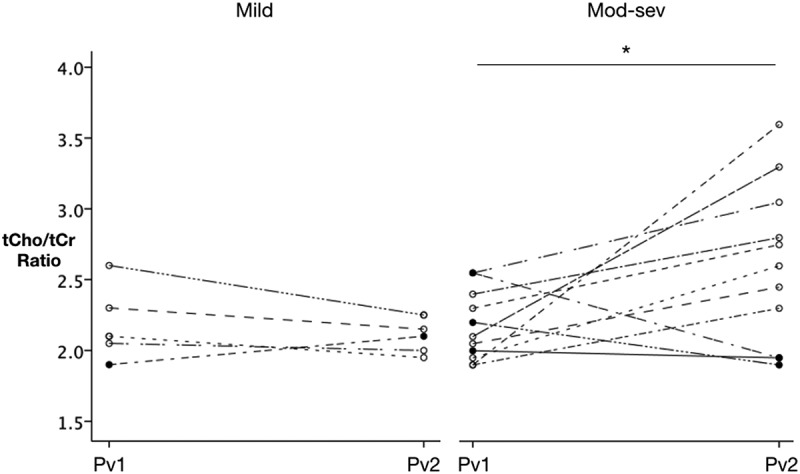
Evolution of total choline ratios from scan 1 to scan 2 in TBI patients. Scatter plots displaying the evolution of total Cho/total Cr ratios in patients between scan 1 (within 24 h) and scan 2 (7–15 days after injury) with mild injuries. There was no significant fall in tCho/tCr ratio in patients in the mild group. There was a significant increase in the tCho/tCr ratio in the moderate-severe group overall (F = 5.54, p = 0.030). Within this group, three patients experienced a fall in tCho/tCr ratio (patients 1, 13 and 15). Despite being classified as moderate-severe at initial presentation, these three patients all improved to a GCS of 15 and had been discharged home within 7 days of their injury. Pv1 = Patient visit 1, Pv2 = Patient visit 2. Within the moderate to severe group, a negative correlation was found between progression of the tCho/tCr ratio and GCS at 1 week (r = −0.77, p = 0.006). The greater increase in tCho/tCr ratio correlated with a poor GCS at 1 week.

We next compared metabolite concentrations measured at both visits across all patients with TBI, and within TBI severity subgroups, to determine the evolution in metabolic disruption between scan 1 (<24 h) and scan 2 (7–15 days). There was no significant difference in metabolite ratios across all patients when considered as a single group. However, distinct patterns of evolution in metabolite disruption emerged when patients were sub-divided according to injury severity. Although no significant changes in metabolite concentrations were observed in the mild TBI group (n = 6), the moderate-severe group (n = 11) exhibited an increase in tCho/tCr ratio between 24 h (scan 1) and 7–15 days (scan 2) following injury (F = 5.54, p = 0.03), although three patients showed a decrease rather than increase in tCho/tCr ratio (Figure 3). When assessed at 1 week, these three patients all had a GCS of 15 and had been discharged home, in contrast to the other eight patients who either remained in patients with a GCS <15 or had significant persistent symptoms. A Spearman’s correlation analysis was performed on the moderate-severe group (n = 11) to further investigate the relationship between total Cho/total Cr ratio change and clinical progression. A negative correlation was found. A tCho/tCr ratio increase between the two scans correlated negatively with a poor GCS at 1 week post injury (r = −0.770, p = 0.006). Sample sizes in each subgroup are very small and this should be considered when interpreting the results.

Analysis of metabolites at individual time points also revealed variation according to severity. At time point 1, although no significant difference was seen at the whole-group level in GLX/tCr ratio, GLX/tCr ratios were lower in the more severely injured (GCS of 3–12) when compared to the less severely injured (GCS of 13–15) (F = 10.16, p = 0.007). After 7 days, this pattern was no longer present. Visual inspection of the tCho/tCr ratios at each time point suggested lower Cho ratios in patients with a mild injury and higher ratios in patients with a more severe injury, however this did not reach significance (Figure 2b).

### White matter tract injury

**Figure 4.**
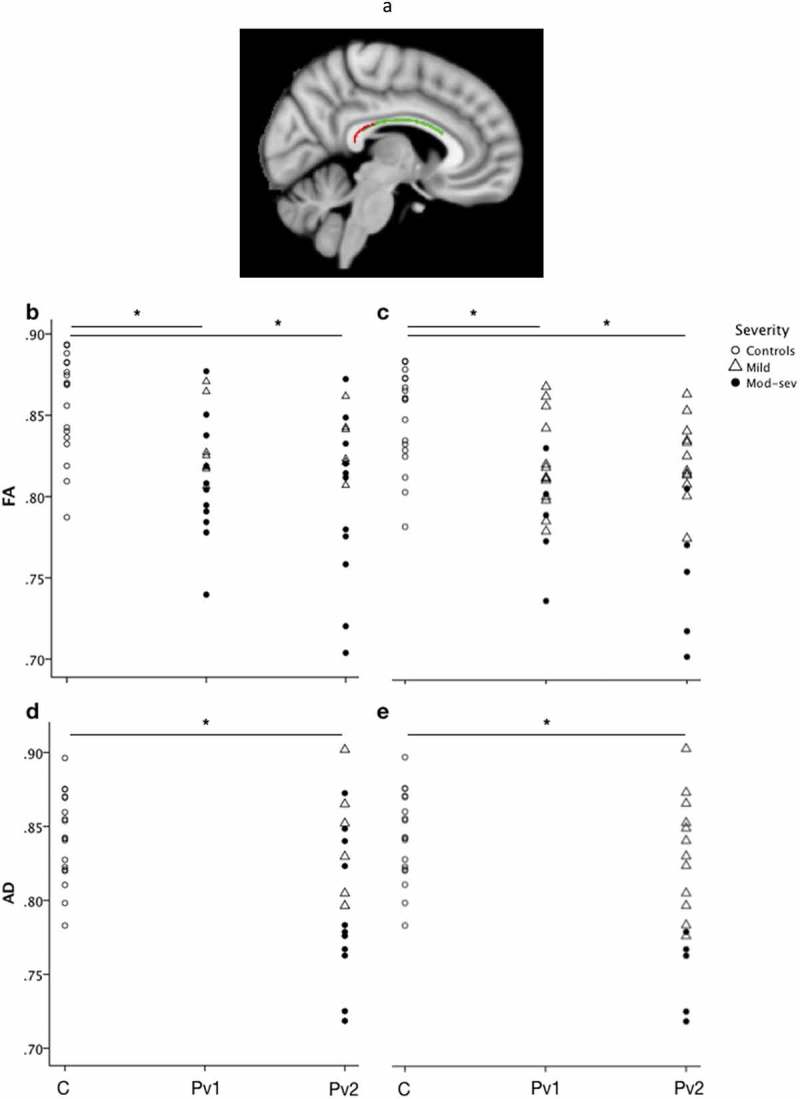
Evolution of white matter parameters following TBI. (a) A sagittal image showing a significant cluster representing FA values lower in patients at scan 1 (<24 h) compared with controls in the splenium of the corpus callosum (p = 0.029). Green represents the corpus callosum part of the mask, red represents the significant cluster. Scatter plot displaying FA (b & c) and AD (d & e) for voxels in the corpus callosum according to severity measured at initial presentation (b & d) and at 1 week post injury (c & e). Using TBSS FA was found to be significantly lower in patients at time point 1 compared with controls (p = 0.029) and in patients at time point 2 compared with controls (p = 0.027). Also, using TBSS AD was significantly lower in patients at time point 2 compared with controls (p = 0.013). AD was not significantly lower at time point 1 (within 24 h of injury) therefore not plotted. The significant cluster is overlaid on an MNI152 image. C = Controls, Pv2 = Patient visit 2. * represents a value for p < 0.05.

To determine whether patients with TBI also show disruption in white matter microstructure of the corpus callosum and cingulum bundle, diffusion parameters measured at scan 1 (<24 h) and scan 2 (7–15 days) were compared against diffusion parameters measured in controls (1 scan only). At the group level, FA was significantly lower in patients compared with controls in the splenium of the CC within 24 h of injury (permutation testing, p = 0.029) (Figure 4). At scan 1, no significant difference was found for AD, RD or MD. At scan 2, both FA and AD were significantly lower in patients compared with controls (p = 0.027 and p = 0.013, respectively) (Figure 4). No differences were found for RD or MD.

To determine whether diffusion measures evolved between the hyper-acute and acute phase in our patient cohort, we performed longitudinal TBSS analyses testing for linear effects in FA, MD, RD and AD *difference* over time between scan 1 and scan 2. Longitudinal analyses revealed increases in both MD and RD between scan 1 and 2 across patients with TBI at the group level (p = 0.028 and p = 0.043, respectively). No significant difference was seen for FA or AD using this method.

To explore whether these differences in microstructural parameters were common to all patients, or might differentially reflect injury severity, we extracted the mean FA, MD, RD and AD from the respective clusters showing significant between-group (FA, AD) white-matter changes, and plotted these for the TBI subgroups for visualization purposes (Figure 4). These summary values were plotted separately for patients according to severity assessed either by GCS at presentation or GCS at one week. When subdivided according to initial GCS, no clear pattern in the evolution of FA or AD was apparent between the two time points. However, when the patients were grouped according to severity assessed at 1 week, mild and moderate-severely injured appeared to segregate, with the lowest FA and AD at scan 2 observed in the more severely injured (Figure 4). No statistical comparison of these subgroups was performed due to the small number of patients still classified as mild after one week (n = 5).

**Figure 5.**
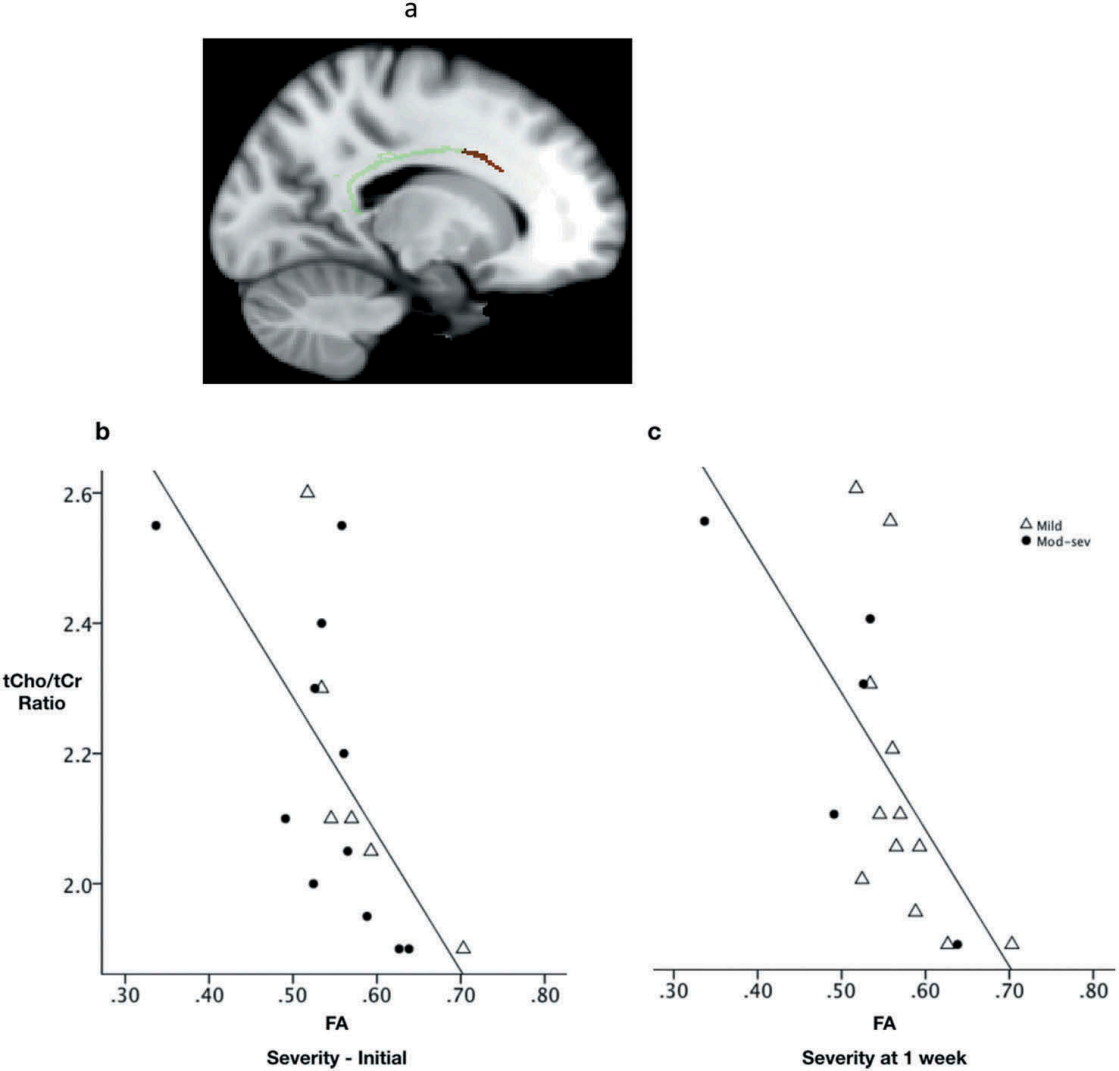
Relationship between FA and total choline/total creatine ratio. (a) Significant cluster from voxel-wise correlation analysis showing lower FA values in the body of the corpus callosum correlating with higher tCho/tCr ratio values in the posterior cingulate cortex measured within 24 h of injury (p = 0.029). For illustrative purposes, scatter plots allow visualization of the relationship between FA from the significant cluster and the mean tCho/tCr ratios for each patient averaged across the whole cluster and input into SPSS to display the correlation. b) Plotted with subgroups according to initial injury severity and c) severity assessed at 1 week.

**Figure 6.**
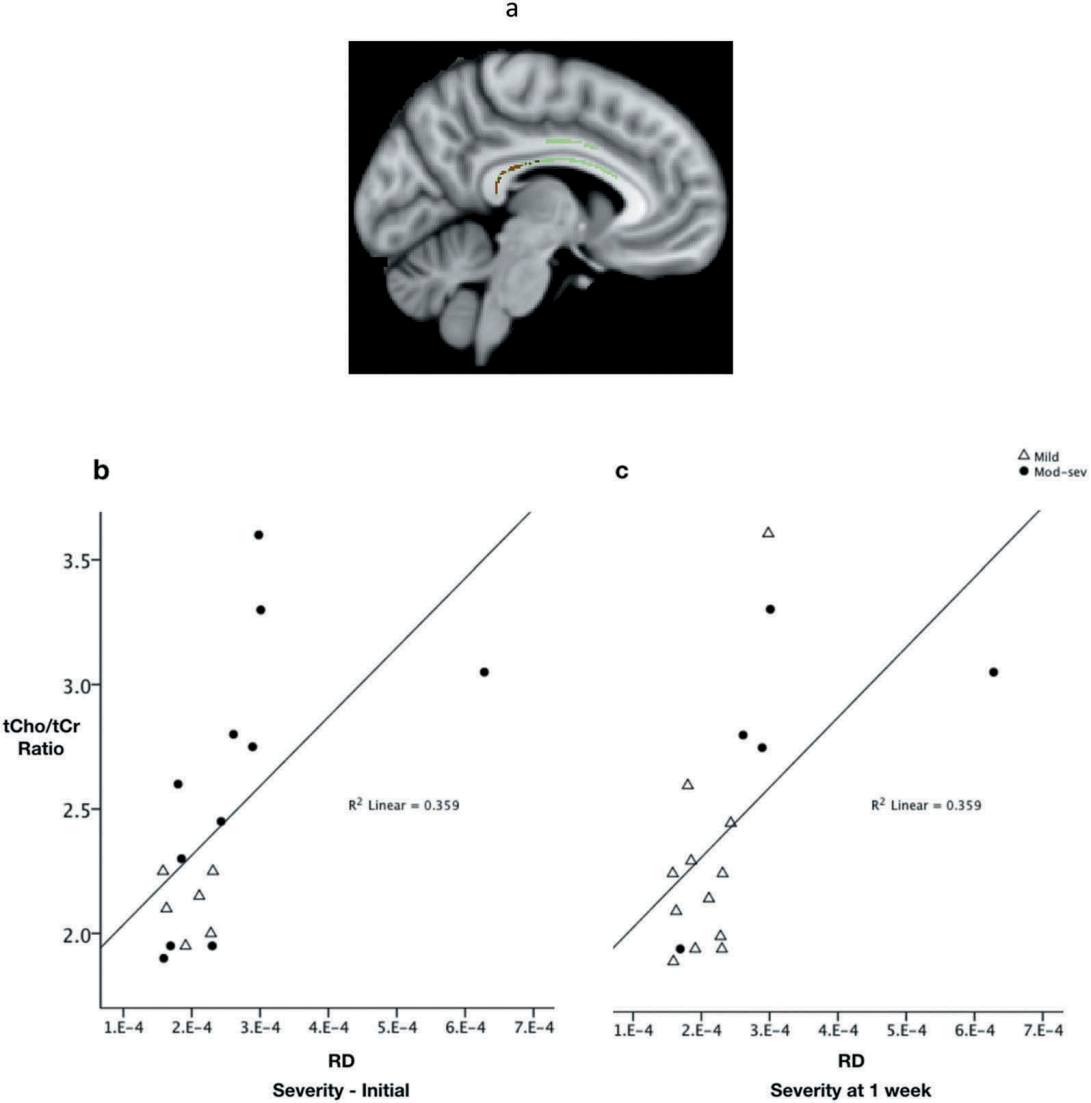
Relationship between RD and total choline/total creatine ratio. (a) Significant cluster showing higher RD values in the body of the corpus callosum correlating with higher tCho/tCr ratio values in the posterior cingulate cortex measured within 24 h of injury. For illustrative purposes, scatter plots allow visualization of the relationship between RD from the significant cluster and the mean tCho/tCr ratios for each patient (p = 0.006) averaged across the whole cluster and input into SPSS to display the correlation. b) Plotted with subgroups according to initial injury severity and c) severity assessed at 1 week.

Finally, we tested for relationships between metabolite ratios in the PCC and CC microstructural properties in patients at each scan as well as change over time, and in healthy controls. No relationships were found between metabolite ratios and diffusion parameters in the healthy controls. In patients within 24 h of injury (scan 1), lower FA values in a cluster localized to the body of the CC were correlated with higher tCho/tCr ratios in the PCC (p = 0.029) (Figure 5b and c). No significant relationship was seen between the other metabolites and diffusion parameters at scan 1. In patients at scan 2 there was a tendency for an increase in RD in the splenium/body of the CC to reflect an increase in tCho/tCr ratio (p = 0.06) in the PCC. No relationship was seen between other metabolites and diffusion parameters at scan 2. When correlating the change in the microstructural parameters with the change in metabolite concentrations among patients, a large change in RD was associated with a large change in tCho/tCr ratio; in other words, progression in RD was positively correlated with progression in tCho/tCr (p = 0.006) (Figure 6).

## Discussion

Our understanding of the secondary injury cascade, and progression of diffuse axonal injury, in the first few hours and days following TBI, is limited by clinical tools that, currently, are not able to reveal the underlying pathophysiological processes thought to take place in injury-susceptible midline brain regions. Beyond the consequence of the primary injury that occurs on impact, the secondary metabolic cascade is central to the progression of injury and DAI. Here we set out to investigate, firstly, whether such metabolic disruption would be evident in the posterior cingulate cortex (PCC) within hours of trauma and reflects disruption in markers of axon coherence in the associated corpus callosum (CC) and cingulum bundle (CB). Secondly, we characterized progression in MRI measures of metabolite ratios and white matter microstructure from the hyper-acute (<24 h) to acute (7–15 days) phase after TBI of varying severities. We found MRS evidence of neuronal injury and possible membrane turnover in the PCC within 24 h of trauma across a range of severities. Metabolic disruptions appeared to progress as the secondary injury cascade evolved between the hyper-acute (hours) and acute phase (days to weeks). Studies in rats provide evidence that RD reflects de/dysmyelination (56,57). Here we identified white matter microstructural damage in the CC within 24 h of injury, with worsening RD, providing evidence for de/dysmyelination across the first 7–15 days post-TBI in humans. Our findings provide indications that the metabolic disturbance created by the secondary injury cascade creates an environment where DAI may progress, reflected by diffusion parameters in the CC. Of clinical interest, both MRI techniques differentiated patients according to injury severity, most importantly reflecting clinical outcome at 1 week.

Progressive posterior cingulate cortex (PCC) metabolite disruption between the hyper-acute and acute phase of TBI

Previous studies using MRS in patients with TBI have found reduced NAA in the parietal lobe, motor cortex, prefrontal cortex and corpus callosum (58–61) in the acute phase (days) and subacute phase (weeks) after injury (62). NAA reduction in the weeks and months following injury appears to reflect long-term outcome (11,63). Animal studies have provided evidence of reduced NAA as soon as 1 h following fluid percussion injury, possibly due to increased NAA utilization in axonal repair processes (64). Depression and subsequent recovery of NAA were also reported in animal studies over the first 7 days following injury, thought to signify recovery (65–67). In humans, the timescale of metabolic disruption remains largely unknown. Most human studies were performed more than 3 days post injury and there has been speculation that metabolic changes might not be apparent in patients with mild TBI until 3 days post injury (68). Our findings provide evidence of injury in the PCC, in normal-appearing brain, as soon as 3.5 h following trauma, characterized by a fall in NAA and an increase in Cho, which persist through the first 7 days following injury. This marked reduction in NAA in the hyper-acute phase, even in patients with mild TBI, suggests early neuronal injury in the PCC grey matter, while increased Cho, which was evident at this time point and then progressed, suggests the evolution of cell membrane disruption from the hyper-acute to the acute phase.

### Evidence of injury progression and indication of severity

Between scan 1 and 2, tNAA/tCr remained low at the group level in our study, with no evidence of differing patterns according to clinical severity in this time period. The longer-term consequences of these early aberrations in NAA may emerge from ongoing follow-up studies incorporating neuropsychological outcome measures.

In contrast, results from studies investigating Cho have revealed elevated levels in patients post injury (63,69–71) and a correlation with long-term outcome when measured at 7 days in both white matter and grey matter (14). Elevation of Cho following TBI may reflect membrane disruption associated with shearing or stretch injuries seen in DAI as a result of astrocytosis or inflammation (14,71,72). A relationship between choline levels and severity would, therefore, be expected, and indeed the highest elevations in Cho were previously reported in the more severely injured (14). Our data similarly indicate elevated tCho/tCr levels at both time-points in patients compared with controls and further provide evidence for greater progression in patients with more severe injury (compared to mild injury) at scan 2. In the mild group, visual examination of individual patients suggested normalization of tCho/tCr ratios in our sample size within our follow-up period. In contrast, in the moderate-severe group, there was a significant increase in tCho/tCr ratios over time, with notable variations at the single-patient level. In three moderate-severely injured, tCho/tCr levels remained stable or fell within the first 7–15 days after injury (declining to normal levels). Retrospective assessment of these patients revealed that by day 7 they all had a GCS of 15 and had been discharged home, in contrast to the other 8 patients who either remained in-patients with a GCS <15 or had significant persistent symptoms. Although the numbers were small in the moderate-severe group (n = 11), limiting interpretation, a negative correlation was found between the tCho/tCr ratio increase and GCS at 1 week. Consequently, we propose that a progressive elevation in tCho/tCr ratios in the first week following injury may represent the progression of secondary brain injury after the first 24 h and offer a potential index of TBI severity. Small numbers in the subgroup analysis must be taken into account when interpreting the data.

Perhaps surprisingly in our study, GLX did not differ significantly between patients and controls at the group level. Previous studies suggest glutamate plays a crucial role in TBI (73). Acute post-traumatic glutamate release is responsible for excitotoxicity leading to neuronal injury, cell death and dysfunction of surviving neurons (73). Delayed disruption of excitatory glutamate circuits leads to deficits in cognitive and motor function (73). The pathophysiological consequence of glutamate excitotoxicity is similar from mild (43) to severe TBI (74). MRS studies using a controlled cortical impact model in mice demonstrate decrease in glutamate at 2 and 4 h after injury (12) and increase in pericontusional glutamine (75) with variability in the respective progression of glutamate and glutamine over subsequent days.

We did not find a significant difference in GLX/tCr ratios between controls and patients at either time points. While patients with moderate-severe injury showed low GLX/tCr ratios at scan 1 (within 24 h of injury), there were no longer any differences between severity subgroups at scan 2 (7–15 days). A pattern in GLX/tCr ratio was seen regarding severity at the first scan, with low ratios in moderate-severely injured, but not at the second scan, possibly reflecting normalization at 1 week. We speculate that reduced GLX/tCr ratios may reflect early glutamate reduction associated with diffuse injury in the moderate-severe group with apparent normalization occurring due to development of increase glutamine associated with contusions in these patients.

### Diffusion evidence of progressive corpus callosum (CC) injury

The posterior cingulate cortex (PCC) sits adjacent to, and has efferent connections with (76), the splenium of the corpus callosum (CC) which is frequently impacted in trauma (20,21). The cingulum bundle (CB) provides structural connection between the PCC with the medial temporal lobes and the ventromedial prefrontal cortex (27). Although we used a mask of the CB and CC, significant disruption to diffusion parameters was only found in the CC. Imaging evidence of disruption in the CC offers a sensitive correlate of injury severity (22). The relationship between injury in the PCC and white matter tract injury in the CC in the hyper-acute phase may provide important insight into the diffuse metabolic environment that predisposes patients to the progression of diffuse axonal injury (DAI). No studies, to our knowledge, have investigated longitudinally the relationship between diffusion parameters, measured from the hyper-acute phase to the acute phase, and PCC metabolic injury. Few studies have investigated axonal injury in the hyper-acute phase, and all were conducted either in patients with mild injury or beyond 24 h of injury. Over a longer time period, FA has been shown to decrease and MD increase in patients with severe TBI imaged in the subacute (weeks) to chronic (months) phase following injury (36). Conversely, animal studies of severe TBI using a cortical impact model with DTI and histological analysis have revealed a temporal pattern of variation in diffusion parameters on a much more immediate timescale following injury (41). A lateral fluid percussion model has also been used to provide evidence supporting the use of DTI in the detection of microstructural changes and how these correlate with injury at a cellular level (6). Histological evidence in mice of axonal injury within 24 h correlated with reduced FA, AD and MD, while reactive gliosis detected at 4 days and macrophage infiltration, demyelination and edema after 1 week were associated with elevated RD, AD and MD alongside decreased FA (41). In our study of diffusion changes in the hyper-acute phase following human TBI, we found reduced FA in patients compared with controls within 24 h of injury, which remained low at 7–15 days. While these results indicate reduced white matter microstructural coherence early after trauma, FA is not a specific marker of any individual injury process and did not provide a sensitive marker of injury progression over the first 15 days in our TBI population.

Conversely, we observed a reduction in AD in patients who had suffered TBI compared with controls, specifically at scan 2 (7–15 days after trauma). AD is a measure of diffusivity parallel to the axonal fibres, and a reduction likely reflects damage and degeneration of the axonal fibres (77,78). Axonal integrity would be expected to vary by injury severity (as the degree of axonal disruption may vary) (33,57). However, when we compared patients according to their initial severity at presentation, we found no difference in AD between mild and moderate-severe TBI. When instead, patients were compared according to their status at 7 days, patients with TBI who had a GCS of 14 or less, or experienced persistent symptoms, had significantly lower AD than patients who had been discharged home, had a GCS of 15, with no persistent symptoms. Subdivision of the patient group according to severity measured at initial presentation revealed no significant difference between mild and moderate-severe injury at either scan. However, when the severity was measured again at 7 days, according to GCS, a distinction could be seen in the AD between the two severity groups. AD may, therefore, offer a sensitive marker of injury severity when early clinical and radiological assessment fail to accurately predict short-term outcome. AD could alternatively reflect injury progression and act as a marker of the developing secondary injury cascade. The lack of a significant fall in AD at the first scan may reflect a more prolonged period of injury progression. Imaging multiple times every day would be required to understand the optimal period for intervention that might limit the secondary injury cascade.

Vasogenic oedema and potentially demyelination affect white matter following TBI. Radial diffusivity is thought to reflect diffusion of water perpendicular to white matter fibres (79) which increases in response to both demyelination (56) and dysmyelination (57). MD describes the per voxel average magnitude of water diffusion regardless of diffusion direction (37). Vasogenic oedema following TBI is thought to result in decreased FA and increased MD and RD (37). In our TBI group, RD and MD both increased significantly between the two scans at 24 h and 7–15 days, providing evidence of injury progression but also more specifically of possible demyelination and axonal degeneration. RD has been shown to increase in white matter in the context of pathology and may provide evidence of axonal degeneration and demyelination (80). Therefore, the combination of FA, as an early marker of injury, and RD and MD, providing evidence of oedema or injury progression, may be important in characterizing development and evolution of DAI within the first days and weeks after injury.

### Relationship between injury in the posterior cingulate cortex (PCC) and the corpus callosum (CC)

Examining the relationship between our observed abnormalities in metabolite ratios and in microstructural tissue properties following TBI, we firstly found that high tCho/tCr ratios in the PCC were associated with low FA in the CC as early as 24 h following TBI. At 7–15 days, there was a tendency for increased RD values to mirror elevations in tCho/tCr ratios. As previously mentioned, elevated Cho provides evidence of membrane damage and/or repair following TBI (13,15). The relationship of FA and RD with tCho/tCr, at the two different time points, respectively, suggests early evidence of injury to the PCC and consequent downstream injury to the CC. The grey matter injury and white matter disruption may be linked due to anatomical connection or by being subject to global injury that we see represented in the PCC, the effects of which manifest in the CC with diffuse axonal injury. It is possible that the tCho/tCr elevation in the PCC precedes the RD increase. We could speculate that the tCho/tCr elevation signifies membrane disruption in the midline structures and offers a mechanism for axonal degeneration/demyelination in the CC that emerged over the first 7–15 days following trauma. Longitudinal assessment of diffusion parameters in the CC over weeks to months could improve our understanding. Nonetheless, our observed relationship between metabolic disruption and white matter injury in separate but related anatomical regions, within the normal-appearing brain on conventional imaging, suggest that metabolic disruption that takes place in the context of diffuse axonal injury may provide information about the likelihood of secondary injury progression.

### Limitations

Limitations exist with this study. Wide heterogeneity exists in our cohort including age, injury type, initial severity and outcome. While there are benefits to assessing a range of injury severities, the lack of homogeneity in other areas raises some challenges regarding interpretation of results. In addition, due to the challenges associated with scanning intubated, ventilated, severely injured patients, in the first few hours after trauma, the sample size was limited. Greater patient numbers are needed to replicate our findings and better understand the relationship between metabolite and diffusion parameter variation and progression of the pathophysiological secondary injury cascade. Larger cohorts for further subgroup analysis of mild, moderate and severe TBI are needed to further explore the potential for MRI as a tool to provide markers of severity in the hyper-acute phase. In our study, we were limited by the time intervals between scans. More frequent scanning sessions within the first 7 days would provide a more detailed understanding of the temporal window for evolution of the secondary injury cascade. While these limitations lead to the potential for variable interpretation of the results, our study does highlight the utility of MRS and DTI to assess injury severity and progression of the secondary injury cascade from the first few hours following trauma across all severities.

In conclusion, in this longitudinal study of patients who had suffered TBI of all severities, we identified evidence of metabolic injury and white matter tract injury within 24 h of trauma. We found evidence of prolonged membrane disruption in the PCC and progression of DAI in the CC, beyond the first 24 h, with metabolite ratios acting as potential markers of worsening severity. In addition, diffusion parameters seem to provide a more accurate prediction of a patient’s short-term outcome than conventional assessment with initial GCS. To our knowledge, a relationship between metabolite ratios in the PCC and diffusion parameters in the CC has not been shown before in the hyper-acute phase, and suggests a link between damage to the PCC and the CC, specifically the splenium, which may characterize the progression of DAI and potentially development of damage to functional networks. The vital next step will be to identify a relationship between markers of injury in the PCC and CC in the hyper-acute phase, and long-term clinical and neuropsychological outcome. Additionally, further investigation of the functional networks, thought to underpin cognitive deficits following TBI, may provide evidence of the link between early metabolic disruption in the PCC and long-term clinical outcome.

Identifying the biochemical disruption that underpins the development of DAI, in the time frame that corresponds with progression of the secondary injury cascade (yet to be defined in humans), may create the opportunity to implement treatment to prevent its progression.
